# Editorial: Advances in artificial intelligence and robotics in joint arthroplasty

**DOI:** 10.1186/s42836-025-00302-5

**Published:** 2025-04-03

**Authors:** Andrew P. Kurmis, Sébastien Lustig, Francesco Zambianchi, Yunsu Chen

**Affiliations:** 1 College of Medicine & Public Health, Flinders University, Bedford Park, SA 5042 Australia; 2 Discipline of Medical Specialties, University of Adelaide, Adelaide, SA 5005 Australia; 3 Department of Orthopaedic Surgery, Lyell McEwin Hospital, Elizabeth Vale, SA 5112 Australia; 4 Orthopaedic Surgery and Sports Medicine Department, Croix-Rousse Hospital, Lyon University Hospital, 69317 Lyon, France; 5 Department of Orthopaedic Surgery, Azienda Ospedaliero-Universitaria Di Modena, University of Modena and Reggio-Emilia, 41125 Modena, Italy; 6 Department of Orthopedics, Shanghai Sixth People’s Hospital, Shanghai Jiao Tong University, Shanghai, 200233 China

**Keywords:** Artificial intelligence, Robotics, CPAK, Technology-assisted surgery, Machine learning

## Abstract

The recently-completed special edition of *Arthroplasty* entitled 'Advances in Artificial Intelligence and Robotics in Joint Arthroplasty', brings together novel and innovative research from around the world in this cutting edge topic area. With robotics, artificial intelligence and technology-assistance (inside and out of the operating theatre) all becoming increasingly relevant to contemporary practice, we hope the readership will find this special edition an informative and thought-provoking read. Ultimately twelve individual papers were accepted for the edition, covering a range of exciting and novel applications. There clearly exists an ongoing need to provide further validation of new applications and, in many instances, replication of results away from designer sites is needed to provide robust generalizability of use. While several of the included papers show wide international collaboration, the prospect of future interactive work engaging leaders and think-tanks regionally and globally provides a tantalizing opportunity. With global health settings under increasing pressure and scrutiny to provide greater provision of joint replacement services – with the expectation of even more predictable (better) outcomes in a cost and resource efficacious manner – necessity will continue to drive further work exploring how technology-incorporation into arthroplasty care pathways might help address many of these considerations. There are undoubtedly exciting times ahead.

## Introduction

With ever-increasing patient expectations of enduring function and quality-of-life preservation, there exists an ongoing pressure to seek newer and better ways to diagnose and manage individuals with disabling joint pathology. Internationally, most contemporary estimates predict a rapidly increasing demand for joint replacement surgery in the coming two decades [1]—a need that will likely far exceed existing surgical capacity. Current means will need to evolve towards newer / novel approaches to meet the rising demand in a climate whereby cost and resource accountability (including sustainability), and consistent achievement of lasting, high functional, standards will be paramount.

In many realms, technology-assisted surgery has already shown potential to improve multiple aspects of the arthroplasty patient journey—from initial diagnosis through to medium-term (and possibly longer-term) post-operative functional outcomes. Both artificial intelligence (AI) applications and the use of intra-operative robotics are exciting areas of active development and application. While they have generated much enthusiasm (and marketing), there is a need for scientists and clinicians alike to ensure that the evidence base that underpins the use of such technologies stays ahead of the enthusiastic hype [2]. While cutting-edge work in surgical robotics aims to improve the precision and performance of operative plans (and hence patient outcomes and satisfaction) on an individual patient level, these complex and highly sophisticated machines bring their own unique set of challenges including training and learning curves, alterations to existing surgical techniques and workflows, and often the considerable associated expense with purchase and ongoing upkeep.

AI has already been explored in a wide range of applications relevant to the care of arthroplasty patients from predictive diagnostics and imaging interpretation to patient selection and educational metrics, to administrative and cost-funding considerations, to augmented operative planning, and the predictive utility for a number of medical (and non-medical) outcomes [2, 3]. Concerns have been raised previously about the wider generalizability of much of the published AI literature to date, and a lack of reproduction of key findings away from algorithm designer sites or highly specialized quaternary centers.

## Background

In 2020, *Arthroplasty* launched its first targeted special edition—“Artificial intelligence in joint Arthroplasty”. Guest co-edited by Professor Yan Wang (Editor-in-Chief of *Arthroplasty*) and Dr. Quanbo Ji, the collection was highly-successful and generated much professional interest. Driven by support from contributing authors and experts in the field, reviewers of the journal, and—most importantly—the wide international readership, the editorial team of *Arthroplasty* resolved to commission a follow up special edition. With international invited guest editors including Andrew Kurmis (Australia), Sebastien Lustig (France), Francesco Zambianchi (Italy) and Yunsu Chen (China) an initial call for papers was released in late 2023—under the title “Advances in Artificial Intelligence and Robotics in Joint Arthroplasty”. It was hoped this would provide an opportunity to attract cutting edge research across an exciting range of topics covered by this novel domain. The article collection was closed by the journal in December of 2024, with 12 high-quality articles ultimately accepted for inclusion in the special edition.

The primary goal of the special issue of *Arthroplasty* was to provide an opportunity for clinical researchers from across the globe to contribute to the advancement of knowledge in the area of contemporary arthroplasty, specifically relating to robotic and AI applications. We sought to bring together a number of high-quality works in these fields to serve as both an informative and educational platform, but also to strengthen the foundation of science that supports the use of these exciting new technologies. All works accepted for publication underwent a rigorous peer review process, prior to further consideration for inclusion. We hope the special issue will be both updating and thought provoking and will provide a foundation contribution of knowledge to many of the areas within the realm of AI applications and technology-assisted arthroplasty.

### Scope and specific themes

Within the gazetted scope of publication intent, we encouraged diversity of content to include both basic science and clinical research spheres. Original clinical research, structured (systematic) reviews and proof-of-concept papers were all invited and considered. Topic prompts included, but were not limited to:

- Advancements in robotic technologies (including novel applications) and the clinical evidence that might support wider uptake.
- Cost and outcome analyses.
- Head-to-head comparisons between the performance and outcomes of arthroplasty surgery and either conventional approaches and/or computer-navigated methods.
- Applications with demonstrated benefit to patient-reported outcome measures (i.e., PROMs).
- Registry-level evidence of the performance and survivorship of robot-assisted arthroplasty procedures.
- Robotic applications in complex primary and revision surgery.
- The use of AI in optimized implant prediction/templating.
- The role of AI in improving patient outcomes.
- AI utility in optimized patient selection pathways.
- Demonstration of whole episode-of-care cost savings through the establishment and implementation of AI technologies.
- The clinical outcomes of the use of validated AI applications in non-specialist centers (i.e., reflecting more generalizable use).

### Summary of the included articles

The editors were most pleased at such a positive response from both local and international authors (and authorship groups), with submitted manuscripts canvassing a broad range of relevant sub-topics. Applications involving intra-operative utilization of semi-active surgical robotics featured highly—perhaps reflecting clinical interest and contemporary practice demands—and AI applications were also represented. Utilization of these advanced technologies in both hip and knee arthroplasty environments were included. The following considers and summarizes the key intent and outcomes of each of the included papers.

Originating from Australia, the work of Gieroba and colleagues (2023) [4] explored the post hoc, arithmetic calculation of hip-knee-angle (aHKA) using computed tomographic (CT)-based image captures otherwise collected routinely as part of the standard Stryker Mako® (Kalamazoo, MI, USA) total knee arthroplasty (TKA) planning acquisition series. The HKA forms an integral part of the pre-operative planning assessment for many knee surgeons and is often used to inform peri-operative decisions regarding target implant component positioning. Using a recruited cohort of 68 patients undergoing primary TKA, the work contrasted the accepted international convention of standardized, standing long-leg alignment radiographs (LLR) and a conventional mechanical HKA (mHKA) determination, with the CT-based comparator (aHKA). The authors report several potential advantages to the CT-based approach including “more reproducible” measurement of key angular indices (i.e., the medial proximal tibial (MPTA) and lateral distal femoral (LDFA) angles), and the capacity to overcome some of the intrinsic barriers to accurate measurement including inconsistent patient positioning during image capture, inflexible joint contractures and challenging body habitus. Of practical relevance, the lack of a demonstrated effect of observer seniority with measurement precision or repeatability suggests the results reported may have wide practical generalizability. With high levels of reported correlation with the gold standard approach, the authors reasonably conclude that aHKA measurement assessment from the CT protocol may obviate the need for the routine performance of LLRs.

The international, collaborative work presented by Edelstein and colleagues (2024) [5] reported the potential value of intra-operative measurement determination of coronal plane alignment of the knee (CPAK) classification derived from imageless robotic TKA applications. Using the OMNI-botics system (Corin, Cirencester, UK) they present results based on a cohort of 62 TKA procedures performed for osteoarthritis (OA) and contrast the classification consistency with the accepted LLR standard. As secondary considerations, the authors also explored whether utilization of the generic 2 mm wear assumption (Nav_lit_) or the more contemporary optimized wear assumption (Nav_opt_) resulted in more accurate CPAK classification versus the LLR “gold standard”. The MTPA, LDFA, joint line obliquity (JLO) angle and the aHKA were independently recorded and compared for each patient. The authors report that in 94% of cases the Nav_opt_ method permitted accurate CPAK determination within one classification group when contrasted with the LLR approach. Utilising the Nav_lit_ method this precision dropped to 88%, albeit with a mean measurement error of just 0.6° for all requisite parameters. Based on these findings, the paper concludes that the imageless robotic navigation approach can be reliably employed to calculate CPAK parameters for arthritic knees undergoing uncomplicated TKA, with good agreement to the LLR standard. As with the previous work of Gieroba et al. (2023) [4], this finding suggests the independent acquisition of standing LLRs may be unnecessary in the standard workflow of planned TKAs using these widely-available robotic platforms.

Continuing the interest and clinical relevance of the CPAK classification, the work of Agarwal and colleagues (2024) [6] report an important retrospective study exploring the impact of change in constitutional CPAK grouping when performing TKA and correlate this with metrics of patient satisfaction using conventional PROMs. The authors set out to provide objective evidence to challenge the widely-accepted dogma that patient dissatisfaction after TKA can be linked to CPAK group change from the pre- to post-operative state. Generated using the ROSA® semi-active robotic system (Zimmer Biomet, Warsaw, IN, USA), the authors report their results from 134 patients undergoing robot-assisted TKA utilising a cementless implant combination and a mechanical alignment balancing philosophy (target HKA 0°). Key pre-operative CPAK measurement determinants and JLO were established using standard LLRs and post-operatively from the CT scout images obtained as part of a standardized metal-artifact reduced Perth CT protocol. The findings suggest 125 of 134 included patients (93.28%) were “happy” with the outcome of their surgery, as determined by PROMs recorded at one year after surgery. The CPAK classification was changed in 116 patients (86.57%) and therefore maintained in just 18 (13.43%). Based on these results, the paper concluded that the widely-accepted 15%–20% of patients dissatisfied after primary TKA surgery cannot be explained by post-operative change in the patient’s native joint line or CPAK classification.

Moving to total hip arthroplasty (THA) applications of contemporary robotics, Marcovigi and colleagues (2024) [7] report on the impact of proximal femoral geometry upon THA stem position using the Stryker Mako® (Kalamazoo, MI, USA) system and the “enhanced” workflow protocol. Originating from Italy, the authors present the outcomes of 102 consecutive patients undergoing routine THA for end-stage OA, utilising the straight, single-wedge, Accolade® II femoral stem (Stryker; Mahwah, NJ, USA). Recognising the widely-accepted philosophy of combined component target anteversion of 25°–45° (as popularised by Dorr et al. (2009) [8]), the paper acknowledges the potential challenges associated with component target attainment when using uncemented femoral stems (noting the limited surgeon capacity to purposefully alter position based on host bone geometry). All included patients underwent a standardized CT-based pre-operative scanning protocol permitting determination of a series of associated measurement indices, including femoral neck version (FNV), anterior and posterior cortex anteversion, and femoral metaphyseal axis anteversion—each at three different anatomic levels. Using the highly-precise measurement capacity of the intra-operative robotic system, the authors report a mean native FNV of 6.6°. The mean final stem version reported was 16.4°. The results presented suggest that femoral anteversion progressively increases from the upper neck region to the proximal metaphysis. With the goal of achieving final stem position within the 5°–25° anteversion range (with an “ideal” target of 15°), the authors suggest aligning the stem to the native femoral anteversion at a level 10 mm above the lesser trochanter. They report that doing so reliably achieved target version in 96.1% of included cases.

The collaborative work of Buchan and colleagues (2023) [9] retrospectively explored objective differences in post-operative narcotic consumption in patients undergoing primary THA when contrasting utilization of robotic-assistance versus conventional instrumented methods. Other than use of the robot, all other surgical considerations were standardized. All included procedures were performed using an anterior approach and an intra-operative, fluoroscopy-assisted, technique. Robotic-assisted cases were performed using the ROSA® Total Hip System (Zimmer CAS, Montreal, QC, Canada). In total, 211 patients were included in the study. Mean analgesic requirements using morphine milligram equivalents (MME) were compared between the two groups for both the in-hospital and post-hospital discharge periods (up until 6 weeks post-surgery). Patient-reported pain data were also reported using visual analogue scores. The findings reported suggest significant comparative reductions in post-operative analgesic consumption (i.e., MMEs) using the robotic-assisted technique when compared to the conventional instrumented standard. The author’s highlight the value of their result given the competing relevance of rapid recovery / short-stay arthroplasty pathways and the safe need for optimized pain control in the early post-operative period. As the author’s themselves rightly concede however, the exact mechanism through which the use of robotic-assistance in THA leads to lesser degrees of post-operative pain remains to be fully elucidated and undoubtedly provides an opportunity for future investigation.

Addressing a highly-relevant and widely-discussed issue, Yee and colleagues (2024) [10] from China present an interesting prospective paper attempting to quantify the reliability and reproducibility of pre-resection ligament tension assessment using an imageless, robot-assisted, TKA approach. Using data captured using the CORI TKR system (Smith & Nephew, USA) the authors present their findings collected from a small, controlled, cohort of 24 knees. Accurate and reproducible determination of the state of ligament tension (as a surrogate for “balance”) remains a key consideration of the performance of primary TKAs, whether performed using technology-assistance or not. Poor achievement of a “balanced” knee has been associated with post-operative patient dissatisfaction and higher subsequent revision rates. The authors rightly highlight that the intuitive “feel” that a senior arthroplasty surgeon relies upon in making definitive decisions about intrinsic ligament tension is an entirely subjective construct, one often difficult to “teach” to others. The use of intra-operative sensor devices (manual or electronic) is often helpful in providing quantitative tension measures but can usually only be applied after at least preliminary bony cuts have been performed. Access to reliable “pre-cut” ligament balance data may allow the surgeon to undertake distal femoral and proximal tibial bone cuts best permitting final overall balance. The paper reports the intra- and inter-rater reliability in performing ligament tension assessment. The key findings of the study suggest that application of the robotic technology affords at least “good-to-excellent” reliability in assessing all knee tension positions, with the exception of intra-rater determination of the “flexion lateral” state. The most reliably measured knee position for ligament tension assessment was the medial compartment in extension. An interesting secondary consideration was the finding that more senior arthroplasty surgeons consistently produced larger gaps during knee balance assessment in both the medial and lateral compartments in nearly all measured flexion angles. It will be interesting to see this work reproduced using other commonly-available robotic-knee systems to understand the translatability of these preliminary results.

Another well-published Australian research hub present their work assessing mid-flexion instability after uncomplicated TKA using intra-operative pressure sensors. Armendariz and colleagues (2024) [11] report a novel clinical analysis cross-checking final (i.e., surgeon accepted) knee balance. Acknowledging that prosthetic ‘instability’ persists as a major indication for revision surgery, the authors explore the prevalence of quantifiable mid-flexion instability after completion of primary TKAs using robotic-assistance. Combining the Mako® robotic-assisted TKA system and the Verasense (OrthoSensor, FL, USA) digital pressure measurement tool, the group report their findings from a cohort of 72 knees undergoing uncomplicated TKA. An *a priori* determination was made regarding acceptable soft tissue “balance”, represented by a difference in measured compartment pressures of 15 pounds (i.e., 6.8 kgs) of force (or less), representing local clinical practice standards. The key findings of the paper showed no significant difference between measured pressures in the medial compartment when recorded at 10°, 45° and 90°, but statistically significantly higher pressures in the lateral compartment at 10° (*P* < 0.001)—albeit still not reaching the 15-pound pressure threshold. When specifically contrasting the pressures of both compartments at 45° (versus the 10° and 90° degree positions) no patient showed a pressure difference of more than 15 pounds. The authors conclude that there was no clinical (or statistical) evidence of mid-flexion instability when performing Mako®-assisted primary TKAs using a single radius, cruciate-retaining prosthesis.

Zheng and colleagues (2024) [12] report their kinematic evaluation and early clinical outcomes of a preliminary cohort of TKAs performed utilising the patient customized iTotal CR TKA (Conformis, MA, USA) implanted with technology-assistance using TiRobot Recon Robot (TINAVI, Beijing, China). The novel combination allows utilization of enhanced, smart tool, intra-operative assessment features including gap quantification, compartment force determination, and live femoral-tibial tracking. The authors report a mean processing time for custom implants of just 6 weeks. A total of 17 knees were included in this small initial assessment cohort. The authors introduce the term ‘differential’ TKA to describe the surgical workflow employed, driven by objective real-time data informing a mathematical approach to procedural performance. Again, highlighting a need to better address the 15%–20% of patients routinely dissatisfied with their primary TKA, the authors propose that the use of patient-specific implants, advanced intra-operative assessment tools, and the execution capacity of a modern TKA robot will lead to improved functional and patient satisfaction outcomes. Based on the small sample size, the authors conclude that the reported implant and technology combination permits attainment of ‘excellent’ intra-operative joint kinematics including gap balancing, compartment pressures and relative component motion through range, with acceptable radiographic and early clinical outcomes (13 “very satisfied” knees, 3 “satisfied” and one patient describing their early result as “neutral”). Of relevance, the authors suggest the described system may be of future value in specific patient demographics (such as certain Asian populations) where the typically-encountered knee morphotype does not necessarily well-match conventionally-available “off-the-shelf” implant sizes. Further research reporting the results from larger cohort sizes, and with longer-term follow up, is now indicated to continue this preliminary work.

Fary and colleagues (2023) [13] present the outcomes of a prospective, multicentre cohort analysis, assessing immediate post-operative range-of-movement (rom) gains comparing 216 robot-assisted TKAs with 216 propensity-matched control patients whose TKAs had been performed using manual instrumentation. Early rom attainment is often considered a sign of a well-performed TKA and has been associated with improved PROMs. The previously-reported correlation between 3 month rom and patient satisfaction, quality-of-life measures, and 12 month rom were considered in the study design. The authors set to test the hypothesis (and widely held viewpoint) that the higher degree of bony cut precision achievable using robotic techniques leads to improved early patient active rom. Cohorts were propensity-matched by age, gender, BMI and general comorbidities. Pre-operative active rom measurements were compared with one month and three months post-operative equivalents. All robotic TKAs were performed using the ROSA® semi-active robotic system (Zimmer Biomet, Warsaw, IN, USA), and received one of three widely used Zimmer Biomet knee prosthesis options. The key findings suggest significant improvements in rom using the robotic technology at both one and three months (with mean values of 6.9° and 4.9° degrees better than those seen in the instrumented cohort). Patients included in the robotic grouping also had a higher odds ratio (2.15) of achieving 90° of flexion at one month. The authors conclude that, compared with instrumented equivalents, robotic TKA patients achieved faster recovery of active rom to three months post-operatively—noting that change in rom, rather than absolute rom achieved, have previously been associated with functional, patient satisfaction and quality-of-life metrics. The authors justly acknowledge the limitations of their study, including patient, surgeon and location heterogeneity which may confound the strength of the final reported results. Nonetheless, this work adds valuable foundation evidence to support claims of the ability of robotic-assisted TKAs to permit earlier rom engagement and range attainment.

The final clinical paper of the special edition reported on the novel extension of robot-assisted surgery to revision TKA applications. The paper by Ngim and colleagues (2023) [14] originates from the Epworth Musculoskeletal Clinical Institute in Victoria, Australia and describes the early follow up (to 18 months) of 19 patients undergoing revision TKA utilising the Stryker Mako® (Kalamazoo, MI, USA) robotic system. Recognising the challenges often associated with revision TKA, the authors present a roadmap of pre-, peri- and post-operative considerations when managing such patients and explore how current generation robotics may value-add to this process. The mixed inclusion cohort included 12 primary TKAs, 4 UKAs, and three 1st to 2nd stage revisions for previous periprosthetic injection (PJI). They describe use of the Mako® robot and techniques for overcoming issues with in situ metalwork at the time of pre-operative CT scanning (i.e., primary implants), baseline bony surface registration, and how best to preserve host bone stock while maintaining an anatomic joint line. The high-quality paper describes the successfully employed technique in a clear and logical way that facilitates replication by others. While acknowledging the preliminary nature of the work the authors provide an exciting insight into future extension of robotic technologies and provide some measure of confidence in consideration for doing so based on the reported high-quality outcomes. The need for dedicated modifications to existing robotic software pathways to facilitate dedicated use in revision surgery is highlighted as is the need for modified pre-operative image acquisition. The challenge of selecting consistent and reproducible implant and/or bony landmarks for intra-operative surface registration remains to be best defined.

The final two included papers in the special edition explore the exciting realm of incorporation of AI technologies into routine arthroplasty practice. With the potential value of AI in surgery having been widely championed, the transition from theoretical use to real world use requires sensible and thoughtful validation and oversight to ensure that both the incredible potential of such technologies is optimally capitalized and to ensure the evidence base supporting the use of AI continues to meet rigorous scientific standards. Both included papers delve into the current state of understanding through detailed reviews of the current literature base. The paper by Chong and colleagues (2023) [15] describes a structured PRISMA review of AI applications in the area of PJI prevention. Focusing on machine learning (ML) applications, the paper identifies 11 key studies for analytic inclusion, all reported to have a ‘fair’ grade of methodologic quality as per the NIH quality assessment tool. Divided into the categories of PJI “prediction”, “diagnosis”, “antibiotic application” and “prognosis” the authors suggest likely value in capitalizing upon the strengths of ML-based approaches but caution a need for further research to provide sound and robust evidence to permit wider uptake. They present convincing AUC data to support the use of ML in each of the four clinical domains and highlight potential value to treating clinicians in the pooled knowledge effects of access to AI-informed datasets. The consistency and reproducibility of AI diagnostic pathways may lead to improved benchmark standards-of-care.

The final paper in the edition [3] presents an up-to-date meta-synthetic review of the literature base related to AI applications within the specific field of TKA surgery. The paper broadly considers pre-, intra- and post-operative applications and highlights the already demonstrated value of AI technologies in many practical areas. The need to transition published work from “demonstrations of concept” to generalisable and reproducible mainstream applications (with demonstrated external validity) is clear and the opportunity for future related works in this realm are widely indicated. Current AI strengths in TKA application already include mass data handling, outcome prediction, and general administrative tasks where such technologies have likely value in time (and cost) saving and optimizing patient pathway decision making. An awareness that some exciting and promising AI technologies have, so far, failed to better existing human-driven standards is an important reminder to us all to ensure that the evidence base underpinning AI use keeps pace with the rapidly growing international hype.

## Conclusions

The recently-completed special edition of *Arthroplasty* entitled “Advances in Artificial Intelligence and Robotics in Joint Arthroplasty”, was an exciting opportunity to bring together novel and innovative research from around the world in this cutting-edge topic area. With robotics, AI and technology-assistance (inside and out of the operating theatre) all being (or becoming) highly relevant to contemporary practice, we hope the readership will find this special edition an informative and though-provoking read. As the included papers highlight, great work has been achieved in these areas already, but there is much room for ongoing investigation to validate and broaden the key outcomes reported herein and to widen the safe scope of application of these technologies. The potential for interactive and collaborative efforts is tantalizing in bringing together researchers and research teams from across the globe. In a time where all health settings feel increasing pressure to meet provision of arthroplasty care in line with increasing demand, and whereby outcome, cost and resource utilization considerations are paramount, there are certainly exciting times to navigate ahead.

## Data Availability

Not applicable.

## Abbreviations

aHKA: Arithmetic hip-knee angle
AI: Artificial intelligence
AUC: Area under the curve
CPAK: Coronal plane alignment of the knee
CT: Computer tomography
FNV: Femoral neck version
HKA: Hip-knee angle
JLO: Joint line obliquity
LDFA: Lateral distal femoral angle
LLR: Long leg radiograph
mHKA: Mechanical hip-knee angle
ML: Machine learning
MME: Morphine milligram equivalents
MPTA: Medial proximal tibial angle
NIH: National Institutes of Health
OA: Osteoarthritis
PJI: Prosthetic joint infection
PRISMA: Preferred Reporting Items for Systematic reviews and Meta-Analyses
PROMs: Patient-reported outcome measures
THA: Total hip arthroplasty
TKA: Total knee arthroplasty
UKA: Unicompartmental knee arthroplasty
USA: United States of America
